# Increasing precipitation weakened the negative effects of simulated warming on soil microbial community composition in a semi-arid sandy grassland

**DOI:** 10.3389/fmicb.2022.1074841

**Published:** 2023-01-10

**Authors:** Shaokun Wang, Xingchi Jiang, Junyao Li, Xueyong Zhao, Erniu Han, Hao Qu, Xujun Ma, Jie Lian

**Affiliations:** ^1^Urat Desert-Grassland Research Station, Naiman Desertification Research Station, Northwest Institute of Eco-Environment and Resources, Chinese Academy of Sciences, Lanzhou, China; ^2^Key Laboratory of Stress Physiology and Ecology in Cold and Arid Regions of Gansu Province, Lanzhou, China; ^3^College of Resources and Environment, University of Chinese Academy of Sciences, Beijing, China; ^4^Urat National Nature Reserve Management Bureau of Bayannur, Bayannur, China

**Keywords:** global change, warming, increasing precipitation, soil microbial diversity, microbial functional genes

## Abstract

Soil microbial diversity, composition, and function are sensitive to global change factors. It has been predicted that the temperature and precipitation will increase in northern China. Although many studies have been carried out to reveal how global change factors affect soil microbial biomass and composition in terrestrial ecosystems, it is still unexplored how soil microbial diversity and composition, especially in microbial functional genes, respond to increasing precipitation and warming in a semiarid grassland of northern China. A field experiment was established to simulate warming and increasing precipitation in a temperate semiarid grassland of the Horqin region. Soil bacterial (16S) and fungal (ITS1) diversity, composition, and functional genes were analyzed after two growing seasons. The result showed that warming exerted negative effects on soil microbial diversity, composition, and predicted functional genes associated with carbon and nitrogen cycles. Increasing precipitation did not change soil microbial diversity, but it weakened the negative effects of simulated warming on soil microbial diversity. Bacterial and fungal diversities respond consistently to the global change scenario in semiarid sandy grassland, but the reasons were different for bacteria and fungi. The co-occurrence of warming and increasing precipitation will alleviate the negative effects of global change on biodiversity loss and ecosystem degradation under a predicted climate change scenario in a semiarid grassland. Our results provide evidence that soil microbial diversity, composition, and function changed under climate change conditions, and it will improve the predictive models of the ecological changes of temperate grassland in future climate change scenarios.

## 1. Introduction

Global change, such as warming and precipitation fluctuation, directly and indirectly, alters vegetation (Rupp et al., 2000; Walker et al., 2006; Del Grosso et al., 2008; Bjorkman et al., 2018) and soil microbiota (Nottingham et al., 2019; Rillig et al., 2019; Brigham et al., 2021; Sun et al., 2021). As a consequence of increasing greenhouse gases due to land use change and fossil fuel consumption, global climate warming has been recorded worldwide (IPCC, 2013), and global mean temperature is predicted to increase by 2–7°C at the end of this century. Temperature is a primary driver of biological processes, and climate warming has impacted biodiversity significantly in all kinds of terrestrial ecosystems (Chen et al., 2011). Many studies have revealed that climate warming has negative effects on soil microbial biomass (Suzuki et al., 2016; Verbrigghe et al., 2022), diversity (Wu et al., 2022), community composition (Guo et al., 2018), and function (Xue et al., 2016). Precipitation is one of the most important factors influencing microbial community composition in terrestrial ecosystems (Zhou et al., 2018). Increasing precipitation is beneficial for plant growth (Hou et al., 2021; Zhu et al., 2022) and arthropod diversity (Liu et al., 2020) in arid and semiarid areas. A large number of studies have been carried out on the impact of increased precipitation on soil microbial community composition (Huang et al., 2015). However, the soil microbial community responded differently to increasing precipitation in different ecosystems. Most of the studies were related to soil microbial biomass and diversity and rarely referred to microbial function (Zeglin et al., 2013; Xu et al., 2020). Although many studies have been carried out to reveal how single and co-joint global change factors affect soil microbial biomass and composition in terrestrial ecosystems (Xue et al., 2016; Zhou et al., 2020; Marín and Kohout, 2021; Rodriguez-Ramos et al., 2021; Verbrigghe et al., 2022; Wu et al., 2022), it is still unexplored that how soil microbial diversity, composition, and function respond to increasing precipitation, warming, and their interaction in a semiarid grassland of northern China.

Soil microbes are fundamental in litter decomposition, soil formation, and plant growth by controlling the biogeochemical cycles (Van Der Heijden et al., 2008; Chapin et al., 2011; Jacoby et al., 2017). They are very sensitive to environmental changes and could respond rapidly to temperature and precipitation changes (Zhang et al., 2013; Waldrop et al., 2017). Key steps of carbon and nitrogen cycles are mostly driven by soil bacteria or/and fungi (Zhou et al., 2012; Nelson Albright et al., 2016).

The temperature and precipitation are highly fluctuating during the past 50 years and the climate is predicted to be warmer and wetter in the Horqin region of northern China (Zhao et al., 2000; Shi et al., 2003; Liu et al., 2011). The Horqin region is located in the southeastern part of Inner Mongolia. It is a typical semiarid temperate grassland for studying desertification and restoration due to climate change and human activities (Zhao et al., 2003, 2015). This original landscape of the Horqin region was tree-scattered (mainly elms, *Ulmus* spp.) grassland. This area had suffered from desertification due to climate change and unsustainable land-use practices before the 1970s, and the savanna-like grassland shifted into mobile sand dunes. However, the Chinese government carried out the Fencing and Non-grazing Policy since the 1970s, and the policy was implemented gradually in the Horqin region. The enforcement of the policy has accelerated the recovery of the degraded sandy grassland into fixed dunes and grasslands after decades of restoration (Zhao et al., 2015; Wang et al., 2022).

Based on previous studies on the responses of soil microbial communities to climate changes in the semiarid area, a field experiment was conducted to simulate warming, increasing precipitation, and their interaction to explore the impact of global change on soil microbial diversity, community composition, and function in a semiarid temperate grassland of northern China. We hypothesized that (1) simulated warming will decrease soil microbial diversity, dominant microbial phyla, and functional genes, (2) increasing precipitation will weaken the negative effects of warming on soil microbial community composition, and (3) soil bacterial and fungal communities will respond differently to global change scenarios. To test these hypotheses, soil bacterial (16S) and fungal (ITS1) genes were sequenced by the Illumina MiSeq platform, and their alpha and beta diversity, community composition, and functional genes associated with carbon and nitrogen cycles were analyzed in the treatments of warming, increasing precipitation, and their interaction.

## 2. Materials and methods

### 2.1. Study area

**FIGURE 1:**
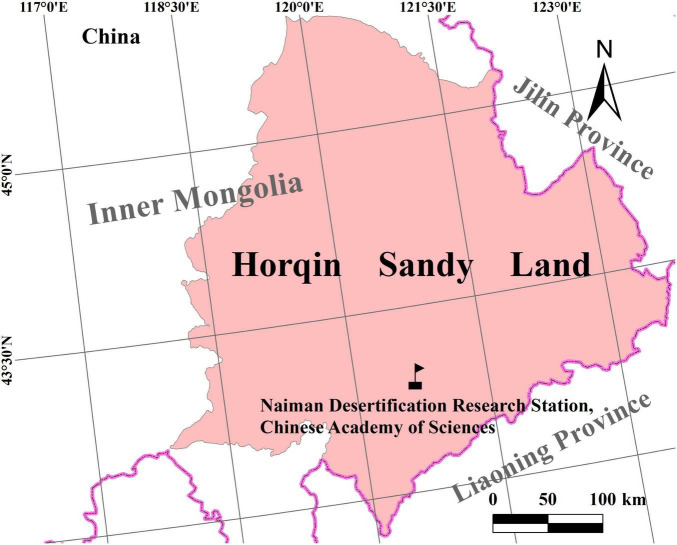
Study area of the Horqin region.

The study was conducted in the Naiman Desertification Research Station of the Chinese Academy of Sciences (short for Naiman Station, 42°55′50′′N, 120°41′51′′E; altitude 360 m), located in Naiman County, southwestern part of Horqin region, Inner Mongolia, northern China (Figure 1). The climate in this area is temperate, semiarid continental monsoonal. The average annual precipitation is 360 mm during the past 50 years, and over 85% is in May to September during the growing season. The annual mean open-pan evaporation is around 1,935 mm, over 5 times more than precipitation. The annual mean temperature is 6.4°C, ranging from a monthly average minimum of −16.8°C in winter to a maximum of 23.5°C in summer. The soil is characterized as sandy chestnut, light yellow in color, sandy in texture, and loose in structure, leading to its vulnerability to wind erosion. The native plant species include *Artemisia halodendron* Turcz., *Lespedeza davurica* L., *Caragana microphylla* L., *Agriophyllum squarrosum* L., *Corispermum macrocarpum* L., *Setaria viridis* L., *Pennisetum centrasiaticum* Tzvel., *Cleistogenes squarrosa* L., and *Chenopodium acuminatum* L. in the region.

Over 70% of the degraded grasslands have been fenced in this area, and the study area has been fenced without grazing for 22 years. The grassland in the study area was dominated by a perennial herb (*P. centrasiaticum*) and a leguminous (*L. davurica*), accompanied by *C. squarrosa*, *S. viridis*, and *C. macrocarpum*, which represent the original natural vegetation in the Horqin region. The overall coverage of the study site was 72.54%, and the above-ground and blow-ground biomass were 193.58 and 134.66 g/m^2^, respectively.

### 2.2. Experimental design

Restored sandy grassland was chosen to conduct the manipulative experiment in the village of Yaoledianzi near Naiman Station. Six 100 m × 100 m sites were selected, and four flat 1 m × 1 m quadrats were randomly set in each site. Open-top glass chambers were built in two of the quadrats in each site to conduct global warming manipulation (T and TW treatment). The open-top glass chamber was hexagonal, with a diameter of 1.2 m and a height of 0.6 m. Plastic shelters were vertically buried at the depth of 0–40 cm under the open-top chambers and the quadrats conducted precipitation manipulation into the soil to avoid side infiltration. The manipulated treatments were increasing temperature (T), increasing precipitation (W), their interaction (TW), and without any treatment as control (CK).

Surface soil temperature was recorded inside the chamber and at the open field, and the temperature was 1.33–4.8°C (mean: 2.64°C) higher inside. According to the past 50-year observed precipitation data in Naiman Station, the mean annual precipitation (MAP) is 343 mm, with 60–80% occurring during the growing season from May to August, and the 10% highest annual precipitation is 467–561, accounting for 37–65% more than the MAP. Therefore, we increased the 50% precipitation during the growing season for the W treatment. We added 50% of daily precipitation (if there are any) in the W and TW plots after each rainfall event. The experiment was conducted in early May 2015. Soil samples were collected at the end of the growing season in early September 2016. The experiment was performed for 2 growing seasons.

A pooled sample was derived from five cores at the depth of 0–10 cm in each chamber as well as the quadrat without chamber using a 3 cm diameter soil auger. Altogether 72 soil samples (4 treatments × 6 sites × 3 replicates) were obtained and stored in sterile plastic bags and kept in a portable icebox during transportation to the laboratory. Soil (carbon, nitrogen, pH, and electrical conductivity) and vegetation (cover, species richness, aboveground biomass, and plant height) were recorded and analyzed as environmental factors.

### 2.3. DNA extraction, PCR, and sequencing

Total genome DNA was extracted using a Qiagen PowerSoil DNA KF Kit (Qiagen, Germany) as soon as the soil samples arrived at the laboratory. Three extracted DNA samples from the same sampling quadrat were mixed as one target DNA and stored at −80°C until sequencing. PCR amplification was conducted using the universal primer 515F-907R for bacteria (Yusoff et al., 2013) targeting 16S rRNA of V4 genes, and ITS1F-2043R for fungi (Adams et al., 2013) targeting ITS1 genes, respectively. PCR products were conducted using electrophoretic detection in 2% agarose gel and purified using the AxyPrep DNA Gel Extraction Kit (Axygen Biosciences, Union City, CA, USA) according to the manufacturer’s instructions and quantified using Quantus™ Fluorometer (Promega, USA). Purified amplicons were pooled in equimolar and paired-end sequenced (2 × 300) on an Illumina MiSeq platform (Illumina, San Diego, CA, USA) according to the standard protocols. The primer and barcode from the obtained DNA sequences were cut off to get raw gene sequencing reads and the raw reads were demultiplexed, quality-filtered by Fastp 0.19.6^1^, and merged by FLASH 1.2.11 (Magoc and Salzberg, 2011). High similarity (≥97%) sequences were clustered using UPARSE 11 (Edgar, 2013) and assigned to the same operational taxonomic unit (OTU). The representative sequences for each OTU were annotated and classified based on the Silva database (Release 138^2^) for bacteria and Unite database (Release 8.0^3^) for fungi. PICRUSt 2.2.0 (phylogenetic investigation of communities by reconstruction of unobserved states^4^) was used to analyze the microbial functional genes based on Kyoto Encyclopedia of Genes and Genomes (KEGG; Release 99.1) (Yin and Wang, 2021). The PICRUSt2 was first used to obtain predicted functional genes (Douglas et al., 2020). Then, predicted functional genes associated with carbon metabolism and nitrogen metabolism pathways were captured from the KEGG Orthology (KO) database (Yergeau et al., 2007; Yan and Feng, 2020) using KEGGREST 1.30.1^5^. Finally, carbon fixation genes, including CF1: Reductive citrate cycle (pckA, rbcL, and rbcS), CF2: Reductive acetyl-CoA pathway (IDH1, korA, korB, korD, korG, porA, porB, porC, porD, and ppc), CF3: Reductive pentose phosphate cycle (acsB, acsE, cdhA, cdhB, cdhD, cdhE, cooF, cooS, coxL, coxM, coxS, fdhA, and fdhB), and CF4: Methanogenesis (fwdA, fwdB, fwdC, fwdD, fwdE, fwdF, and fwdG); carbon degradation genes, including CD1: Cellulose (bglA, bglB, bglX, CBH1, and celF), CD2: Hemi-cellulose (abfA, gumG, MAN, xynA, and xynB), CD3: Starch (amyA, cd, and pulA), CD4: Chitin (chiA, chitin deacetylase, chitinase, and putative chitinase), CD5: Pectin (galacturonidase, pectinesterase, and polygalacturonase), and CD6: Cellobiose transport (cebE, cebF, and cebG); and nitrogen cycle genes, including NC1: Nitrogen fixation (anfG, nifD, nifH, and nifK), NC2: Dissimilatory nitrate reduction (napA, napB, narG, narH, narV, nirB, nirD, and nrfA), NC3: Assimilatory nitrate reduction (narB, nasA, nasB, and nirA), NC4: Denitrification (nirK, norB, norC, and nosZ), and NC5: Nitrification (hao) were selected according to previous studies (Yergeau et al., 2007; Zhong et al., 2018; Yan and Feng, 2020). The sequences could be accessed in the National Center of Biotechnology Information (NCBI) database with the SRA BioProject PRJNA891744.

### 2.4. Data analysis

Soil microbial α-diversity [Chao1 richness, Shannon index, and phylogenetic diversity (PD) index] was calculated using mothur (Schloss et al., 2009). Significant differences among soil microbial alpha diversity (Chao1, Shannon, and PD) in the four treatments were assessed by ANOVA and LSD tests at *p* < 0.05. Microbial β-diversity was calculated by principal coordinates analysis (PCoA) using the Bray–Curtis distance. Significant differences were assessed by ANOSIM at *p* < 0.05 (Rickbeil et al., 2014). The Kruskal–Wallis *H* test was used to analyze the differences in microbial composition at different taxa levels among the four treatments by false discovery rate (FDR) and Tukey–Kramer test (Waite and Campbell, 2006). The microbial community composition was graphed using the Circos software^6^ (Krzywinski et al., 2009). Origin 2017 was used to analyze and describe the statistical data, significance tests, and correlations among the treatments. Pearson’s correlation was used to analyze the relationship between soil microbial diversity and environmental factors.

## 3. Results

### 3.1. Soil microbial diversity

#### 3.1.1. α-diversity

**FIGURE 2:**
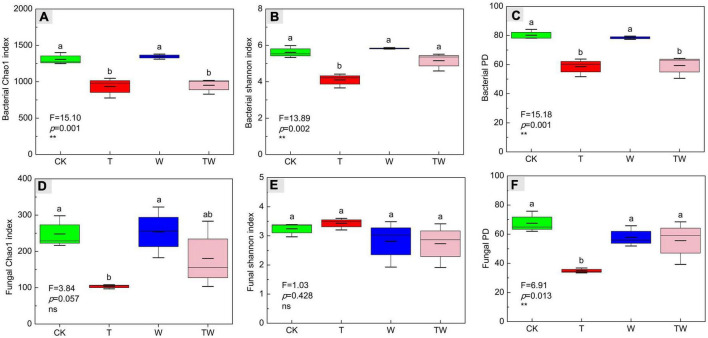
Soil bacterial Chao1 richness **(A)**, Shannon diversity **(B)**, PD diversity **(C)** indices, and fungal Chao1 richness **(D)**, Shannon diversity **(E)**, PD diversity **(F)** indices affected by increasing temperature (T), increasing precipitation (W), and their interaction (TW). Values (mean ± SE) with different letters indicate significant differences at *p* < 0.05 level.

Increasing temperature, increasing precipitation, and their interaction altered soil microbial α-diversity in semiarid sandy grassland (Table 1 and Figure 2). Increasing temperature (T) significantly decreased bacterial Chao1 (Figure 2A), Shannon (Figure 2B), and PD (Figure 2C) indices, and fungal Chao1 (Figure 2D), and PD (Figure 2F) indices. Increasing precipitation (W) significantly affected the bacterial Shannon index. The effects of interaction (TW) on soil microbial α-diversity did not show significant differences from that of control (CK). The results demonstrated that simulated warming decreased soil microbial α-diversity, but increasing precipitation weakened the negative change in a semiarid grassland.

**TABLE 1:** Multifactor ANOVA of bacterial and fungal diversity affected by increasing temperature (T), increasing precipitation (W), and their interaction (TW).

Treatments	Bacteria			Fungi		
	**Chao1**	**Shannon**	**PD**	**Chao1**	**Shannon**	**PD**
T	45.32**	28.09**	45.35**	9.19*	0.03 ns	11.16*
W	0.23 ns	9.44*	0.03 ns	1.34 ns	2.90 ns	1.11 ns
TW	0.04 ns	4.16 ns	0.16 ns	0.99 ns	0.15 ns	4.91 ns

Numbers are *F*-values of multifactor ANOVA. * and ** represent statistically significant differences at *p* < 0.05 and *p* < 0.001, respectively. ns represents not significant.

#### 3.1.2. β-diversity

**FIGURE 3:**
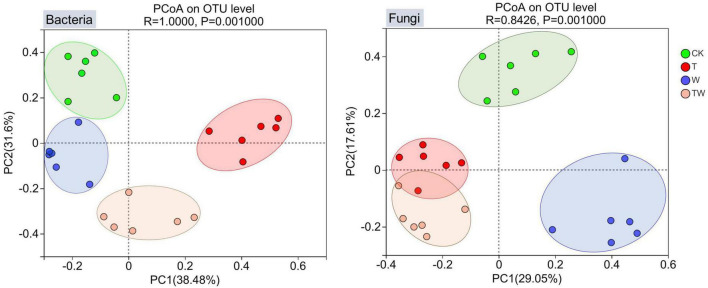
Principal coordinates analysis (PCoA) of soil bacterial and fungal communities. The values associated with axes 1 and axes 2 are the percentage contribution that can be explained by the corresponding axis. CK, control; T, increasing temperature; W, increasing precipitation; TW, their interaction.

Soil bacterial and fungal β-diversities were analyzed by PCoA. PCoA graphs clearly grouped the bacterial and fungal communities according to the simulated treatments (Figure 3). The first two axes of PCoA explained 38.48% (PC1) and 31.60% (PC2) of the total variance for the bacterial community, and 29.05% (PC1) and 17.61% (PC2) of the total variance for the fungal community. The ANOSIM analysis showed that soil bacterial (*p* = 0.001) and fungal (*p* = 0.001) β-diversities were significantly different among the simulated global change treatments.

### 3.2. Soil microbial community composition

**FIGURE 4:**
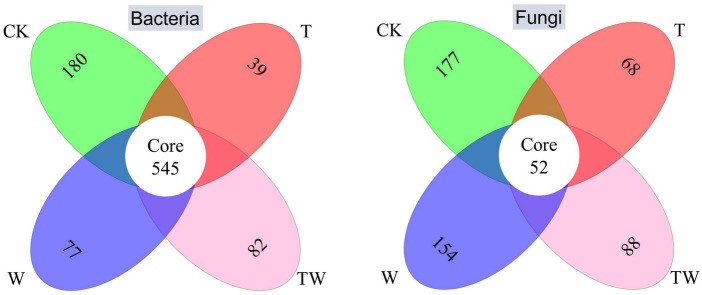
Venn diagram of the soil bacterial **(left)** and fungal **(right)** OTU associated with different treatments. The numbers in different ovals represent the specific OTU in different treatments, and the number of the core represents the common OTU detected in all the treatments. CK, control; T, increasing temperature; W, increasing precipitation; TW, their interaction.

The Venn diagrams showed the numbers of specific bacterial and fungal species in different global change treatments (Figure 4). The core common bacterial species accounted for 37.5–51.9% of the total bacterial species and the specific bacterial species occupied 12.4, 3.7, 5.6, and 7.6% in CK, T, W, and TW, respectively. In comparison, the core common fungal species only accounted for 12.6–25.0% of the total fungal species and the specific fungal species occupied 42.8, 32.7, 39.8, and 31.0% in CK, T, W, and TW, respectively. The proportion of specific fungal species was detected more than that of bacterial species.

**FIGURE 5:**
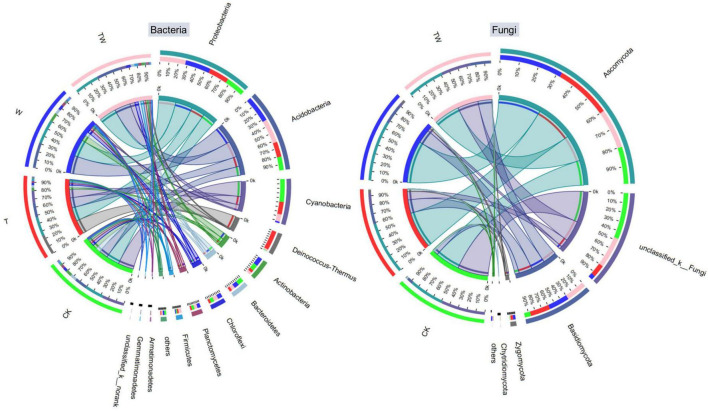
Circos graph of bacterial **(left)** and fungal **(right)** community composition at the phyla level of the different treatments. CK, control; T, increasing temperature; W, increasing precipitation; TW, their interaction.

**FIGURE 6:**
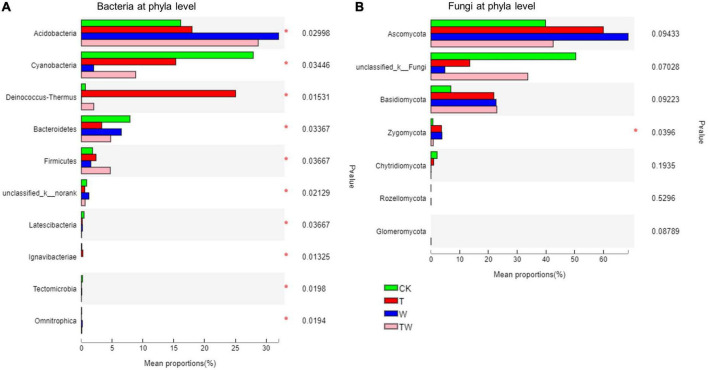
Soil bacterial **(A)** and fungal **(B)** community proportion at the phyla level. (*) Indicates statistically significant differences among the treatments. CK, control; T, increasing temperature; W, increasing precipitation; TW, their interaction.

Soil bacterial and fungal community composition varied significantly among the global change treatments (Figures 5, 6). Soil bacterial communities were dominated by Proteobacteria (27.2%), Acidobacteria (23.6%), Cyanobacteria (13.5%), Deinococcus-Thermus (6.9%), Actinobacteria (6.4%), Bacteroidetes (5.6%), and Chloroflexi (5.0%) at phyla level. Particularly, but not surprisingly, Deinococcus-Thermus, which is tolerant to high temperatures, was the most abundant bacterial phyla in the soil under T treatment. The proportion of Deinococcus-Thermus (25.1%) was higher than Proteobacteria (23.9%) in the T treatment. The Kruskal–Wallis *H* test showed that the bacterial abundant phyla of Acidobacteria, Cyanobacteria, Deinococcus-Thermus, Bacteroidetes, Firmicutes, and the rare phyla of Ignavibacteriae (0.21%), Latescibacteria (0.14%), Tectomicrobia (0.02%), and Omnitrophica (0.01%) were significantly different among the global change treatments (Figure 6A). Soil fungal communities were dominated by Ascomycota (52.7%) and Basidiomycota (18.6%) at the phyla level. The Kruskal–Wallis *H* test showed that only the phylum of Zygomycota (2.25%) presented significantly different among the treatments (Figure 6B).

### 3.3. Microbial functional prediction based on KEGG

**FIGURE 7:**
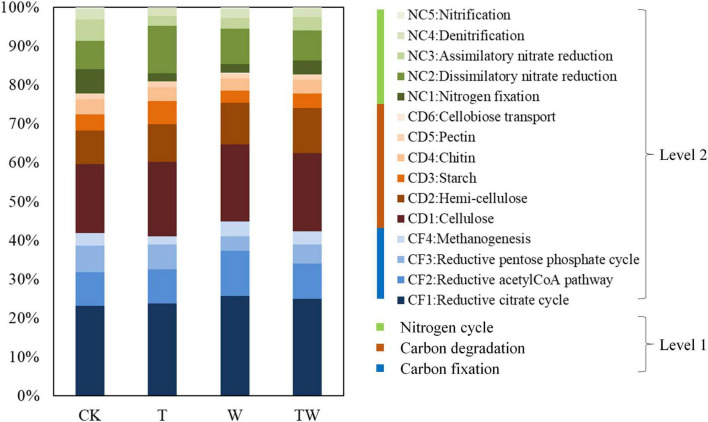
Soil microbial functional genes associated with carbon and nitrogen cycle based on KEGG. CK, control; T, increasing temperature; W, increasing precipitation; TW, their interaction.

The simulated global change altered the abundance of potential functional genes associated with the carbon and nitrogen cycle in the semiarid grassland ecosystem (Table 2 and Figure 7). Increasing temperature (T) decreased the abundance of potential functional genes associated with carbon fixation genes, including the reductive citrate cycle (CF1), reductive acetyl-CoA pathway (CF2), and reductive pentose phosphate cycle (CF3), while it significantly decreased the abundance of potential functional genes associated with methanogenesis (CF4) compared with control (CK). Increasing precipitation (W) significantly increased the abundance of potential functional genes associated with carbon fixation genes, including reductive citrate cycle (CF1), reductive acetyl-CoA pathway (CF2), and methanogenesis (CF4) compared with control (CK). The abundance of potential functional genes affected by TW did not show any statistical significance with those of CK. Increasing temperature (T) did not change the abundance of potential functional genes associated with carbon degradation compared with the control (CK). Increasing precipitation (W) significantly increased the potential functional genes associated with carbon degradation, including cellulose (CD1), hemi-cellulose (CD2), starch (CD3), and cellobiose (CD6) degradation compared with control (CK). Increasing temperature (T) significantly decreased the abundance of potential functional genes associated with nitrogen fixation (NC1), assimilatory nitrate reduction (NC3), denitrification (NC4), and nitrification (NC5), while it increased the abundance of potential functional genes associated with dissimilatory nitrate reduction (NC2) compared with control (CK). Increasing precipitation (W) significantly decreased the abundance of potential functional genes associated with nitrogen fixation (NC1) and assimilatory nitrate reduction (NC3), while it increased the abundance of potential functional genes associated with dissimilatory nitrate reduction (NC2) compared with control (CK).

**TABLE 2:** Soil microbial functional genes associated with carbon and nitrogen cycle in different global change treatments.

Level 1	Level 2	CK	*T*	*W*	TW	*F*
Carbon fixation	CF1: Reductive citrate cycle	22154 ± 1197 b	21878 ± 1215 b	28734 ± 279 a	23668 ± 1999 b	5.8*
	CF2: Reductive acetyl-CoA pathway	8246 ± 1396 b	8136 ± 551 b	13034 ± 612 a	8742 ± 1458 b	4.6*
	CF3: Reductive pentose phosphate cycle	6543 ± 497 a	5801 ± 752 ab	4289 ± 259 b	4658 ± 453 b	3.9 ns
	CF4: Methanogenesis	3148 ± 239 b	1993 ± 405 c	4291 ± 224 a	3288 ± 237 b	10.8**
Carbon degradation	CD1: Cellulose	17072 ± 1457 b	17728 ± 1175 b	22330 ± 89 a	19311 ± 1320 b	4.2*
	CD2: Hemi-cellulose	8207 ± 765 b	8995 ± 1118 bc	12055 ± 133 a	11029 ± 714 ac	5.4*
	CD3: Starch	4026 ± 106 ab	5442 ± 851 a	3466 ± 194 b	3538 ± 74 b	4.3*
	CD4: Chitin	3690 ± 105 a	3358 ± 447 a	3649 ± 74 a	3399 ± 58 a	0.5 ns
	CD5: Pectin	1271 ± 98 a	1324 ± 196 a	1344 ± 39 a	1330 ± 77 a	0.1 ns
	CD6: Cellobiose transport	159 ± 58 ab	34 ± 7 b	247 ± 62 a	61 ± 17 b	5.0*
Nitrogen cycle	NC1: Nitrogen fixation	6008 ± 511 a	1936 ± 605 b	2514 ± 64 b	3333 ± 398 b	16.4***
	NC2: Dissimilatory nitrate reduction	7058 ± 362 b	11259 ± 975 a	10191 ± 106 a	7429 ± 742 b	10.3**
	NC3: Assimilatory nitrate reduction	5281 ± 339 a	2334 ± 498 b	3135 ± 84 b	3276 ± 372 b	12.4**
	NC4: Denitrification	2565 ± 95 ac	1941 ± 60 b	2724 ± 77 a	2075 ± 268 bc	6.3*
	NC5: Nitrification	402 ± 20 a	136 ± 25 b	374 ± 16 ac	303 ± 49 c	15.3**

CK, control; T, increasing temperature; W, increasing precipitation; TW, their interaction. Values (mean ± SE) with different letters in the same row indicate significant differences at *p* < 0.05 level. *, **, and *** represent statistically significant differences at *p* < 0.05, *p* < 0.01, and *p* < 0.001, respectively. ns represents not significant.

### 3.4. Relationship between soil microbial diversity and environmental factors

**FIGURE 8:**
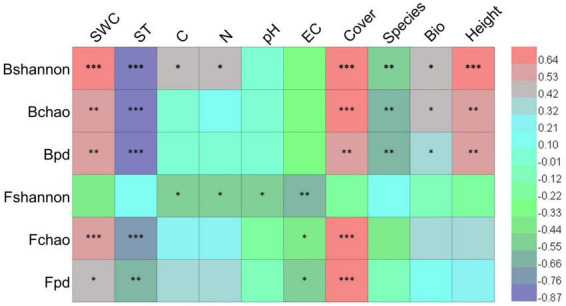
Correlation coefficients between microbial diversity and environmental factors. Bshannon, Bchao, and Bpd represent bacterial Shannon, Chao1, and PD indices, respectively; Fshannon, Fchao, and Fpd represent fungal Shannon, Chao1, and PD indices, respectively; SWC, soil water content; ST, soil temperature; C, soil total carbon content; N, soil total nitrogen content; EC, electrical conductivity; Cover, vegetation cover; Species, plant species richness; Bio, aboveground biomass; Height, plant height. *, ^**^, and ^***^ represent significantly correlated at *p* < 0.05, *p* < 0.01, and *p* < 0.001, respectively.

Pearson’s correlation analysis showed that bacterial and fungal diversity indices were significantly correlated with soil and vegetation factors (Figure 8). Soil bacterial Shannon, Chao1, and PD indices were positively correlated with soil water content, vegetation cover, aboveground biomass, and plant height while negatively correlated with soil temperature and species richness. Soil fungal Chao1 and PD indices were positively correlated with soil water content and vegetation cover while negatively correlated with soil temperature and electrical conductivity. The Fungal Shannon index was negatively correlated with soil carbon, nitrogen, pH, and electrical conductivity. The results suggested that vegetation plays a stronger role in shaping bacterial diversity than fungal diversity under simulated global change conditions.

## 4. Discussion

Warming is recorded worldwide since the last century, and it is predicted to be warmer by the end of the 21st century (IPCC, 2013). It is necessary to clarify the potential impact of increasing temperature on terrestrial ecosystems. Several studies have demonstrated that experimental warming could significantly increase plant biomass by 12.3% across the terrestrial ecosystem from a meta analysis (Lin et al., 2010), especially in low-temperature grassland (Suzuki et al., 2016) and clod tundra (Natali et al., 2012). The increasing temperature could accelerate soil nutrient mineralization and plant photosynthetic rates (Rustad et al., 2001), which promotes plant biomass accumulation. However, the response of soil microbiota to warming is negative, reducing microbial biomass in a subarctic grassland (Verbrigghe et al., 2022), decreasing microbial diversity in a temperate grassland (Wu et al., 2022), and altering microbial functional genes in tallgrass prairie (Xue et al., 2016). The results proved our first hypothesis that warming decreased soil microbial diversity, the proportion of dominant microbial phyla, and the number of potential functional genes associated with carbon and nitrogen cycles in a semiarid grassland. Warming reduced soil bacterial diversity indices (Chao1, Shannon, and PD) by about 30%, and reduced soil fungal diversity indices (Chao1 and PD) by over 50% compared with the control (CK) (Figure 2). Warming decreased some of the dominant bacterial taxa (i.e., Cyanobacteria and Bacteroidetes at the phyla level and Subsection III and Nitrosomonadaceae at the family level), while it increased high-temperature-tolerant bacteria (Deinococcus-Thermus) (Figure 5). Warming decreased carbon fixation genes mostly due to the significant decrease in methanogenesis genes. The abundance of the potential functional genes associated with the nitrogen cycle was significantly lower under warming conditions (T) than that of control (CK). The abundance of potential functional genes of nitrogen fixation, assimilatory nitrate reduction, denitrification, and nitrification was significantly restricted by experimental warming (Table 2).

Increasing precipitation will change vegetation features depending on the amount of increased rainfall. The plant species richness, coverage, and aboveground biomass will increase when the increased rainfall reached over 40% in arid and semiarid grasslands (Luo et al., 2017; Zuo et al., 2020; Zhu et al., 2022). A medium precipitation addition (+30%) increased soil microbial biomass but not the microbial composition in a temperate desert ecosystem (Huang et al., 2015). Our result showed that increasing precipitation (+50%) did not change soil microbial diversity. However, increasing precipitation significantly weakened the negative effects of warming on soil microbial diversity, community composition, and function. The number of OTUs was 19.45% lower for bacteria and 47.60% lower for fungi under warming condition (T) than that of control (CK), while TW reduced the differences by 13.52 and 38.86% for bacteria and fungi, respectively (Figure 2). Warming (T) significantly decreased methanogenesis genes (associated with the carbon cycle) by 36.57%, as well as nitrogen fixation, assimilatory nitrate reduction, denitrification, and nitrification genes (associated with the nitrogen cycle) by 67.78, 55.81, 24.30, and 66.09%, respectively. These functional genes did not show significant differences between TW and CK (Table 2). The result proved our second hypothesis that increasing precipitation weakened the negative effects of warming on soil microbial diversity and functional genes.

Bacteria and fungi respond differently to environmental change because of their morphological traits, utilization strategies, and sensitivity to the environment (Chen et al., 2019). A previous study has demonstrated that soil bacterial communities adapted to environmental changes through changes in the proportions of their taxa, while fungi changed their rare taxa in response to the environmental change along a precipitation gradient (Wang et al., 2021). It was also found that bacteria and fungi respond differently to warming, indicating that the bacterial community was significantly affected by short-term warming but the fungal community was not (Sannino et al., 2022). Our results showed that both bacterial and fungal diversities were significantly decreased and affected by warming and they were not affected by increasing precipitation. Although a greater proportion of specific fungal species was detected in global change treatments than bacterial species, the trends of bacterial and fungal diversities respond consistently to global change scenarios in semiarid sandy grassland, which was different from our third hypothesis. However, the reason for the consistent change seems different between bacteria and fungi, mostly due to the differences in their morphological traits, utilization strategies, and environmental sensitivities (Chen et al., 2019). Simulated warming and increasing precipitation directly changed temperature and soil water content, which influenced plant growth. Vegetation factors play a stronger role in shaping bacterial communities than fungal communities (Figure 8). It can be speculated from our study that soil bacterial composition was more likely to be affected by vegetation factors, and fungal composition was more likely to be affected by soil factors. The influence of temperature and water conditions on bacterial and fungal communities may be different.

## 5. Conclusion

This study illustrated that different global change factors affect the soil microbial community in different ways. Simulated warming significantly decreased soil microbial diversity and functional genes, especially in nitrogen cycle genes. Increasing precipitation did not change soil microbial diversity, but it weakened the negative effects of simulated warming on soil microbial diversity, composition, and functional genes. Bacterial and fungal diversities respond consistently to global change scenarios in semiarid sandy grassland, but the reasons were different for bacteria and fungi. Vegetation factors play a stronger role in shaping bacterial communities than fungal communities under global change conditions. The co-occurrence of warming and increasing precipitation will alleviate the negative effects of global change on biodiversity loss and ecosystem degradation under a predicted climate change scenario in a semiarid grassland.

## Data availability statement

The datasets presented in this study can be found in online repositories. The name of the repository and accession number can be found below: NCBI; PRJNA891744.

## Author contributions

SW and XZ conceived and planned the experiment. SW, HQ, JLia, and EH participated in the field and laboratory work. SW performed the experiment and wrote the original manuscript. XJ, JLi, and XM contributed the data analysis and manuscript editing. All authors contributed to the final manuscript.

## Footnotes

1
https://github.com/OpenGene/fastp

2
http://www.arb-silva.de

3
http://unite.ut.ee/index.php

4
https://github.com/picrust/picrust2/

5
https://rdrr.io/bioc/KEGGREST/

6
http://circos.ca

## References

[B1] Adams R. I. Miletto M. Taylor J. W. Bruns T. D. (2013). Dispersal in microbes: Fungi in indoor air are dominated by outdoor air and show dispersal limitation at short distances. *ISME J.* 7 1262–1273. 10.1038/ismej.2013.28 23426013 PMC3695294

[B2] Bjorkman A. D. Myers-Smith I. H. Elmendorf S. C. Normand S. Rüger N. Beck P. S. (2018). Plant functional trait change across a warming tundra biome. *Nature* 562 57–62. 10.1038/s41586-018-0563-7 30258229

[B3] Brigham L. M. Bueno C. De Mesquita C. P. Smith J. G. Sartwell S. A. Schmidt S. K. (2021). Do plant–soil interactions influence how the microbial community responds to environmental change? *Ecology* 103:e03554. 10.1002/ecy.3554 34622953

[B4] Chapin F. S. Matson P. A. Mooney H. (2011). *Principles of terrestrial ecosystem eology.* New York, NY: Springer.

[B5] Chen I. Hill J. K. Ohlemüller R. Roy D. B. Thomas C. D. (2011). Rapid range shifts of species associated with high levels of climate warming. *Science* 333 1024–1026. 10.1126/science.1206432 21852500

[B6] Chen J. Wang P. Wang C. Wang X. Miao L. Liu S. (2019). Fungal community demonstrates stronger dispersal limitation and less network connectivity than bacterial community in sediments along a large river. *Environ. Microbiol.* 22 832–849. 10.1111/1462-2920.14795 31469494

[B7] Del Grosso S. Parton W. Stohlgren T. Zheng D. Bachelet D. Prince S. (2008). Global potential net primary production predicted from vegetation class, precipitation, and temperature. *Ecology* 89 2117–2126. 10.1890/07-0850.1 18724722

[B8] Douglas G. M. Maffei V. J. Zaneveld J. R. Yurgel S. N. Brown J. R. Taylor C. M. (2020). Picrust2 for prediction of metagenome functions. *Nat. Biotechnol.* 38 685–688. 10.1038/s41587-020-0548-6 32483366 PMC7365738

[B9] Edgar R. C. (2013). Uparse: Highly accurate otu sequences from microbial amplicon reads. *Nat. Methods* 10 996–998. 10.1038/nmeth.2604 23955772

[B10] Guo X. Feng J. Shi Z. Zhou X. Yuan M. Tao X. (2018). Climate warming leads to divergent succession of grassland microbial communities. *Nat. Clim. Change* 8 813–818. 10.1038/s41558-018-0254-2

[B11] Hou E. Litvak M. E. Rudgers J. A. Jiang L. Collins S. L. Pockman W. T. (2021). Divergent responses of primary production to increasing precipitation variability in global drylands. *Glob. Change Biol.* 27 5225–5237. 10.1111/gcb.15801 34260799

[B12] Huang G. Li Y. Su Y. G. (2015). Effects of increasing precipitation on soil microbial community composition and soil respiration in a temperate desert, northwestern china. *Soil Biol. Biochem.* 83 52–56. 10.1016/j.soilbio.2015.01.007

[B13] IPCC (2013). *Working group i contribution to the ipcc fifth assessment report climate change 2013, the physical science basis. Final draft underlying scientific-technical assessment.* Stockholm: IPCC.

[B14] Jacoby R. Peukert M. Succurro A. Koprivova A. Kopriva S. (2017). The role of soil microorganisms in plant mineral nutrition—current knowledge and future directions. *Front. Plant Sci.* 8:1617. 10.3389/fpls.2017.01617 28974956 PMC5610682

[B15] Krzywinski M. Schein J. Birol I. Connors J. Gascoyne R. Horsman D. (2009). Circos: An information aesthetic for comparative genomics. *Genome Res.* 19 1639–1645. 10.1101/gr.092759.109 19541911 PMC2752132

[B16] Lin D. Xia J. Wan S. (2010). Climate warming and biomass accumulation of terrestrial plants: A meta-analysis. *New Phytol.* 188 187–198. 10.1111/j.1469-8137.2010.03347.x 20609113

[B17] Liu R. Navon Y. Steinberger Y. Sternberg M. (2020). Effects of rainfall manipulations versus a natural aridity gradient on plant litter *Arthropods* in desert and mediterranean ecosystems. *Appl. Soil Ecol.* 156:103716. 10.1016/j.apsoil.2020.103716

[B18] Liu X. He Y. H. Zhao X. Y. (2011). Characteristics of precipitation in naiman region of horqin sandy land. *Res. Soil Water Conserv.* 18 155–158.

[B19] Luo Y. Zhao X. Zuo X. Li Y. Wang T. (2017). Plant responses to warming and increased precipitation in three categories of dune stabilization in northeastern china. *Ecol. Res.* 32 887–898. 10.1007/s11284-017-1493-9

[B20] Magoc T. Salzberg S. L. (2011). Flash: Fast length adjustment of short reads to improve genome assemblies. *Bioinformatics* 27 2957–2963. 10.1093/bioinformatics/btr507 21903629 PMC3198573

[B21] Marín C. Kohout P. (2021). Response of soil fungal ecological guilds to global changes. *New Phytol.* 229 656–658. 10.1111/nph.17054 33270913

[B22] Natali S. M. Schuur E. A. G. Rubin R. L. (2012). Increased plant productivity in alaskan tundra as a result of experimental warming of soil and permafrost. *J. Ecol.* 100 488–498. 10.1111/j.1365-2745.2011.01925.x

[B23] Nelson Albright M. Martiny A. Martiny J. (2016). Global biogeography of microbial nitrogen-cycling traits in soil. *Proc. Natl. Acad. Sci. U. S. A.* 113 8033–8040. 10.1073/pnas.1601070113 27432978 PMC4961168

[B24] Nottingham A. T. Whitaker J. Ostle N. J. Bardgett R. D. McNamara N. P. Fierer N. (2019). Microbial responses to warming enhance soil carbon loss following translocation across a tropical forest elevation gradient. *Ecol. Lett.* 22 1889–1899. 10.1111/ele.13379 31489760

[B25] Rickbeil G. J. M. Coops N. C. Andrew M. E. Bolton D. K. Mahony N. Nelson T. A. (2014). Assessing conservation regionalization schemes: Employing a beta diversity metric to test the environmental surrogacy approach. *Divers. Distrib.* 20 503–514. 10.1111/ddi.12146

[B26] Rillig M. C. Ryo M. Lehmann A. Aguilar-Trigueros C. A. Buchert S. Wulf A. (2019). The role of multiple global change factors in driving soil functions and microbial biodiversity. *Science* 366 886–890. 10.1126/science.aay2832 31727838 PMC6941939

[B27] Rodriguez-Ramos J. C. Cale J. A. Jr. Simard S. W. Karst J. Erbilgin N. (2021). Changes in soil fungal community composition depend on functional group and forest disturbance type. *New Phytol.* 229 1105–1117. 10.1111/nph.16749 32557647

[B28] Rupp T. S. Chapin F. S. Starfield A. M. (2000). Response of subarctic vegetation to transient climatic change on the seward peninsula in north-west alaska. *Glob. Change Biol.* 6 541–555. 10.1046/j.1365-2486.2000.00337.x

[B29] Rustad L. Campbell J. Marion G. Norby R. Mitchell M. Hartley A. (2001). A meta-analysis of the response of soil respiration, net nitrogen mineralization, and aboveground plant growth to experimental ecosystem warming. *Oecologia* 126 543–562. 10.1007/s004420000544 28547240

[B30] Sannino C. Cannone N. D’Alò F. Franzetti A. Pittino F. Gandolfi I. (2022). Fungal communities in european alpine soils are not affected by short-term in situ simulated warming than bacterial communities. *Environ. Microbiol.* 24 4178–4192. 10.1111/1462-2920.16090 35691701

[B31] Schloss P. D. Westcott S. L. Ryabin T. Hall J. R. Hartmann M. Hollister E. B. (2009). Introducing mothur: Open-source, platform-independent, community-supported software for describing and comparing microbial communities. *Appl. Environ. Microbiol.* 75 7537–7541. 10.1128/AEM.01541-09 19801464 PMC2786419

[B32] Shi Y. Shen Y. Li D. Zhang G. Hu R. Ding Y. (2003). Disscussion on the present climate change from warm-dry to warm-wet in northwest china. *Quat. Sci.* 23 152–164.

[B33] Sun X. Pei J. Zhao L. Ahmad B. Huang L. (2021). Fighting climate change: Soil bacteria communities and topography play a role in plant colonization of desert areas. *Environ. Microbiol.* 23 6876–6894. 10.1111/1462-2920.15799 34693620

[B34] Suzuki M. Suminokura N. Tanami K. Yoshitake S. Tomotsune M. Masuda S. (2016). Effects of long-term experimental warming on plants and soil microbes in a cool temperate semi-natural grassland in japan. *Ecol. Res.* 31 957–962. 10.1007/s11284-016-1386-3

[B35] Van Der Heijden M. G. A. Bardgett R. D. Van Straalen N. M. (2008). The unseen majority: Soil microbes as drivers of plant diversity and productivity in terrestrial ecosystems. *Ecol. Lett.* 11 296–310. 10.1111/j.1461-0248.2007.01139.x 18047587

[B36] Verbrigghe N. Meeran K. Bahn M. Canarini A. Fuchslueger L. Fransen E. (2022). Long-term warming reduced microbial biomass but increased recent plant-derived c in microbes of a subarctic grassland. *Soil Biol. Biochem.* 167:108590. 10.1016/j.soilbio.2022.108590

[B37] Waite T. A. Campbell L. G. (2006). Controlling the false discovery rate and increasing statistical power in ecological studies. *Ecoscience* 13 439–442. 10.2980/1195-6860200613[439:CTFDRA]2.0.CO;2

[B38] Waldrop M. P. Holloway J. M. Smith D. B. Goldhaber M. B. Drenovsky R. E. Scow K. M. (2017). The interacting roles of climate, soils, and plant production on soil microbial communities at a continental scale. *Ecology* 98 1957–1967. 10.1002/ecy.1883 28464335

[B39] Walker M. D. Wahren C. H. Hollister R. D. Henry G. H. Ahlquist L. E. Alatalo J. M. (2006). Plant community responses to experimental warming across the tundra biome. *Proc. Natl. Acad. Sci. U. S. A.* 103:1342. 10.1073/pnas.0503198103 16428292 PMC1360515

[B40] Wang S. Zhao X. Qu H. Lian J. Ding F. Wang F. (2022). Diversity and composition of culturable fungi in horqin sandy land. *Sci. Cold Arid Reg.* 14 109–119.

[B41] Wang S. Zuo X. Awada T. Medima-Roldán E. Feng K. Yue P. (2021). Changes of soil bacterial and fungal community structure along a natural aridity gradient in desert grassland ecosystems, inner mongolia. *Catena* 205:105470. 10.1016/j.catena.2021.105470

[B42] Wu L. Zhang Y. Guo X. Ning D. Zhou X. Feng J. (2022). Reduction of microbial diversity in grassland soil is driven by long-term climate warming. *Nat. Microbiol.* 7 1054–1062. 10.1038/s41564-022-01147-3 35697795

[B43] Xue K. Xie J. Zhou A. Liu F. Li D. Wu L. (2016). Warming alters expressions of microbial functional genes important to ecosystem functioning. *Front. Microbiol.* 7:668. 10.3389/fmicb.2016.00668 27199978 PMC4858606

[B44] Yan R. Feng W. (2020). Effect of vegetation on soil bacteria and their potential functions for ecological restoration in the hulun buir sandy land, china. *J. Arid Land* 12 473–494. 10.1007/s40333-020-0011-z

[B45] Yergeau E. Kang S. He Z. Zhou J. Kowalchuk G. A. (2007). Functional microarray analysis of nitrogen and carbon cycling genes across an Antarctic latitudinal transect. *ISME J.* 1 163–179. 10.1038/ismej.2007.24 18043626

[B46] Yin Y. Wang J. (2021). Predictive functional profiling of microbial communities in fermentative hydrogen production system using picrust. *Int. J. Hydrog. Energy* 46 3716–3725. 10.1016/j.ijhydene.2020.10.246

[B47] Xu S. Geng W. Sayer E. J. Zhou G. Zhou P. Liu C. (2020). Soil microbial biomass and community responses to experimental precipitation change: A meta-analysis. *Soil Ecol. Lett.* 2 93–103. 10.1007/s42832-020-0033-7

[B48] Yusoff M. Z. Hu A. Feng C. Maeda T. Shirai Y. Hassan M. A. (2013). Influence of pretreated activated sludge for electricity generation in microbial fuel cell application. *Bioresour. Technol.* 145 90–96. 10.1016/j.biortech.2013.03.003 23566463

[B49] Zeglin L. H. Bottomley P. J. Jumpponen A. Rice C. W. Arango M. Lindsley A. (2013). Altered precipitation regime affects the function and composition of soil microbial communities on multiple time scales. *Ecology* 94 2334–2345. 10.1890/12-2018.1 24358718

[B50] Zhang N. Liu W. Yang H. Yu X. Gutknecht J. L. Zhang Z. (2013). Soil microbial responses to warming and increased precipitation and their implications for ecosystem c cycling. *Oecologia* 173 1125–1142. 10.1007/s00442-013-2685-9 23736549

[B51] Zhao H. L. Zhao X. Y. Zhang T. H. (2003). *Desertification processes and its restoration mechanisms in the horqin sand land.* Beijing: Ocean Press.

[B52] Zhao H. Zhang T. Cui J. Li Y. (2000). Effect of climatic changes on environment and agriculture in the past 40 years in interlaced agro-pasturing areas of north china-a case study in horqin sand land. *J. Desert Res.* 30 2–7.

[B53] Zhao X. Y. Wang S. K. Luo Y. Y. Huang W. D. Qu H. Lian J. (2015). Toward sustainable desertification reversion: A case study in horqin sandy land of northern china. *Sci. Cold Arid Reg.* 7 23–28. 10.3724/SP.J.1226.2015.00023

[B54] Zhong Y. Yan W. Wang R. Wang W. Shangguan Z. (2018). Decreased occurrence of carbon cycle functions in microbial communities along with long-term secondary succession. *Soil Biol. Biochem.* 123 207–217. 10.1016/j.soilbio.2018.05.017

[B55] Zhou J. Xue K. Xie J. Deng Y. Wu L. Cheng X. (2012). Microbial mediation of carbon-cycle feedbacks to climate warming. *Nat. Clim. Change* 2 106–110. 10.1038/nclimate1331

[B56] Zhou Z. Wang C. Luo Y. (2018). Response of soil microbial communities to altered precipitation: A global synthesis. *Glob. Ecol. Biogeogr.* 27 1121–1136. 10.1111/geb.12761

[B57] Zhou Z. Wang C. Luo Y. (2020). Meta-analysis of the impacts of global change factors on soil microbial diversity and functionality. *Nat. Commun.* 11:3072. 10.1038/s41467-020-16881-7 32555185 PMC7300008

[B58] Zhu Y. Shen H. Akinyemi D. S. Zhang P. Feng Y. Zhao M. (2022). Increased precipitation attenuates shrub encroachment by facilitating herbaceous growth in a mongolian grassland. *Funct. Ecol.* 36 2356–2366. 10.1111/1365-2435.14100

[B59] Zuo X. Cheng H. Zhao S. Yue P. Liu X. Wang S. (2020). Observational and experimental evidence for the effect of altered precipitation on desert and steppe communities. *Glob. Ecol. Conserv.* 21:e864. 10.1016/j.gecco.2019.e00864

